# The Urodele Limb Regeneration Blastema: The Cell Potential

**DOI:** 10.1100/tsw.2010.115

**Published:** 2010-05-31

**Authors:** Kenyon S. Tweedell

**Affiliations:** Professor Emeritus Department of Biological Sciences, University of Notre Dame, Notre Dame, IN, USA

**Keywords:** regeneration, blastema, dedifferentiation, epigenetics, stem cells, progenitor cells, multipotency, pluripotency, reprogramming, cell transformation, transdifferentiation

## Abstract

The developmental potential of the limb regeneration blastema, a mass of mesenchymal cells of mixed origins, was once considered as being pluripotent, capable of forming all cell types. Now evidence asserts that the blastema is a heterogeneous mixture of progenitor cells derived from tissues of the amputation site, with limited developmental potential, plus various stem cells with multipotent abilities. Many specialized cells, bone, cartilage, muscle, and Schwann cells, at the injury site undergo dedifferentiation to a progenitor state and maintain their cell lineage as they redifferentiate in the regenerate. Muscle satellite reserve stem cells that are active in repair of injured muscle may also dedifferentiate and contribute new muscle cells to the limb blastema. Other cells from the dermis act as multipotent stem cells that replenish dermal fibroblasts and differentiate into cartilage. The blastema primordium is a self-organized, equipotential system, but at the cellular level can compensate for specific cell loss. It is able to induce dedifferentiation of introduced exogenous cells and such cells may be transformed into new cell types. Indigenous cells of the blastema associated with amputated tissues may also transform or possibly transdifferentiate into new cell types. The blastema is a microenvironment that enables dedifferentiation, redifferentiation, transdifferentiation, and stem cell activation, leading to progenitor cells of the limb regenerate.

## INTRODUCTION

There are two categories of stem cells found in vertebrates, including mammals. Those that are derived directly from the embryo or germ cells are defined as pluripotent, since they are self-renewing and capable of differentiating into all cell types of the developing organism. Embryonic stem cell (ES) lines are derived from various embryonic stages. A second type of stem cell is derived from reserve cells sequestered in adult tissues. These somatic stem cells are more restrictive in their potential and, depending upon their cell or tissue source, are considered to be multipotent, able to form more than one cell type, usually limited to their familial developmental lineage. Once individual stem cells are committed to a cell lineage, they become progenitor cells with a more restricted differentiation potential that often depends on their microenvironment or niche[1]. There are several parallels with the potency of somatic stem cells and the cells in the urodele limb regenerate. During urodele limb regeneration and blastema formation, many tissues of the amputated stump release cells that return to their partially differentiated progenitor cell state. In addition, some reserve muscle stem cells are stimulated to form progenitor muscle cells, while other multipotent stem cells can form more than one type of a committed progenitor cell.

New methods have developed to circumvent the use of embryonic cells as a source of pluripotent stem cells. The major success came about when pluripotency was induced directly in somatic cells. The fundamental approach involved nuclear reprogramming, whereby differentiated cell nuclei were returned to a simulated embryonic state. The basic procedure transfers a somatic cell nucleus into an enucleated oocyte or fertilized egg by somatic cell nuclear transplantation (SCNT) or nuclear transfer through cell fusion (NT). The cell now has a resident somatic cell nucleus capable of directing pluripotent cell development and many recent variations on the procedure have been successful. Subsequently, many new pathways have evolved to produce plasticity in adult somatic cells[2].

Of these, a technique perfected by Takahashi and Yamanaka[3] was the epigenetic reprogramming of a differentiated somatic cell genome back to a state similar to that of a pluripotent cell. This was accomplished by the introduction of defined groups of transcription factors, Oct3/4, Sox2, Klf4, and the myc oncogene, causing their overstimulation in the host that returned the cell to a state much like that of the embryo. Fibroblasts from mice or humans were transfected by virus carrying the factors and resulted in the production of induced pluripotent stem cells (iPS) capable of forming multiple cells and tissues. Further refinements in the procedure have indicated that different combinations of transcription factors can induce epigenetic remodeling that produce cells capable of contributing to normal embryonic development[2]. Since many of the controlling factors are active in both kinds of stem cells, there is reason to follow their role in the limb blastema as well.

The effects of epigenetic reprogramming and induced pluripotency in somatic stem cells have been examined recently. The molecular mechanisms involved in stem cells and similar events during normal development have been defined as either functional or molecular changes that cells exhibit as their cell fate changes. These restrictions on the genome are reversible and related to epigenetic modifications of the genome[4]. Changes in the transcription factors and DNA demethylation are related to pluripotency gene activation, and it is likely that similar events play a roll in stem cell activation and differentiation in the limb bud blastema.

## Early Embryonic Limb Formation

In the salamander (tailed amphibian), the prospective cells that are capable of forming a forelimb are located in the *limb field*, a large oval of cells just behind the gill and below the pronephric primordium of the tailbud embryo (Fig. 1). The core area consists of mesoderm covered by epidermis, and can be localized in the prior neurula stage (formation of the brain and spinal cord) and tail bud stages as described in Balinsky[5]. The limb field is greater than the ultimate *limb disc* and, if split into parts and transplanted, will regenerate, each part capable of forming a limb. Early transplantation experiments by Harrison[6] indicated that the mesodermal portion of the field is the source of limb determination and can form a limb with any surface epidermis. Each cell in the limb morphogenetic field can form any part of the limb. Furthermore, the limb field can regulate for lost cells or when new, nondetermined tissues are introduced into it. The first sign of limb development is the formation of a limb bud, an eccentrically located disc of mesoderm covered by ectoderm. It arises in the limb field and forms from migrating mesenchyme cells of the lateral plate mesoderm. Later myoblasts migrate from adjacent somites to form the future skeletal and muscle progenitors[7]. The attached limb bud ectoderm remains smooth and nonthickened as SEM and TEM studies by Tank[8] showed that it does not form an apical ectodermal ridge commonly found in other vertebrates, but the epidermis is initially passive and later interacts with the mesenchyme as the limb bud progresses. A comparison of the cell sources for the embryonic limb bud and the regenerating limb indicates many similarities between the two developmental states. For further details of limb bud development, see Carlson[9] and Stocum and Fallon[10].

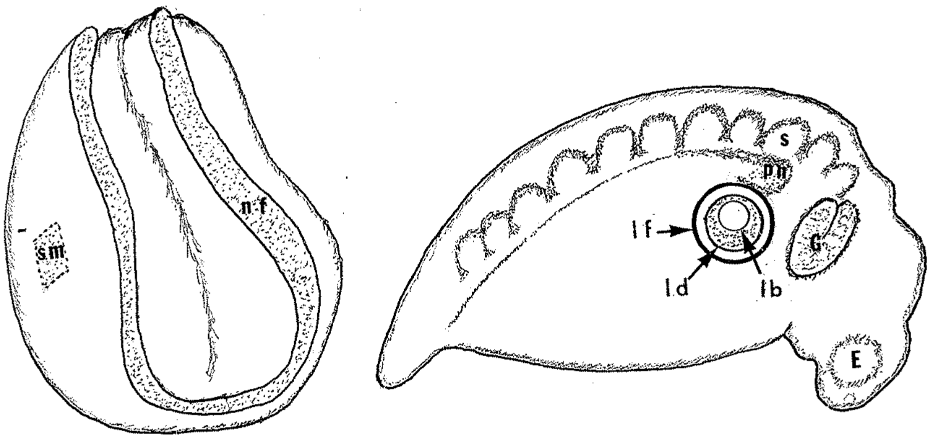

**FIGURE 1.** Normal embryonic salamander forelimb primordial. Left: neurula embryo (stage 15); SM: somitic mesoderm (muscle); nf: neural fold (future brain, bottom). Right: tailbud embryo: forelimb zones; lf: limb field; ld: limb disc; lb: limb bud; S: somite; G: gill; E: eye; pn: pronephros.

## Regeneration

There are certain cell replacement processes in the adult vertebrate that resemble embryonic development. One of these is regeneration, the ability to replace lost tissues, organs, and certain appendages. Many lower vertebrates possess this power, but it is particularly evident in bony fish and amphibia (larval frogs and toads, and adult salamanders and lizards). Different species of amphibia can replace tails and limbs as well as other body components, such as the lens and retina of the eye, jaw, liver, bone, and intestine[11]. The ability to regenerate appendages involves the replacement of several highly specialized cells and tissues, and is related to the transition from the embryonic or larval state to the adult. Anurans (frogs), such as *Rana* and *Xenopus*, can regenerate their tails and early limbs in the larval state, but lose the ability for limb regeneration as they approach metamorphosis into the adult. Adult lizards can only regenerate their tail, but adult newts (salamanders) can replace the missing portions of amputated limbs and tails. Some salamanders display neoteny, incomplete metamorphosis (which varies from species to species); they develop sexually, but some of their somatic tissues remain juvenile, e.g., they retain a cartilaginous skeleton. The success of limb regeneration decreases as development proceeds through the larval, neotenous, and adult states of development, and there are differences between species. Most investigations on limb regeneration have focused on larval, neotenous, and adult forms of tailed amphibian salamanders: *Ambystoma mexicanum* (a neotenic axolotl), *A. punctatum* (spotted salamander), *A. tigrinum* (tiger salamander with environmentally selective neoteny), and the adult newt, *Notophthalmus* (*Triturus) viridescens.* In the urodele (tailed) amphibians, limb regeneration has been studied extensively since it involves a remodeling of many highly differentiated tissues at the amputation site into plastic progenitor cells before regenerating new tissue components of the replaced limb. Known as epimorphic regeneration, the successive developmental stages and controlling factors of limb regeneration in the salamander or newt involves the processes of wound healing, formation of an epidermal cap, histolysis, dedifferentiation, cell proliferation, cell growth, and redifferentiation. Stem cell activation and cell transformation may also take place.

### *A Brief Look at the Sequence of Limb Regeneration*

Following limb amputation in the urodele amphibia, wound healing and closure is accomplished by migration of the epidermis from the adjacent skin over the cut surface from the adjacent skin (Fig. 2). Early investigators of limb regeneration, Polejaiev and then Jeffimoff (reviewed in Rose[12], described

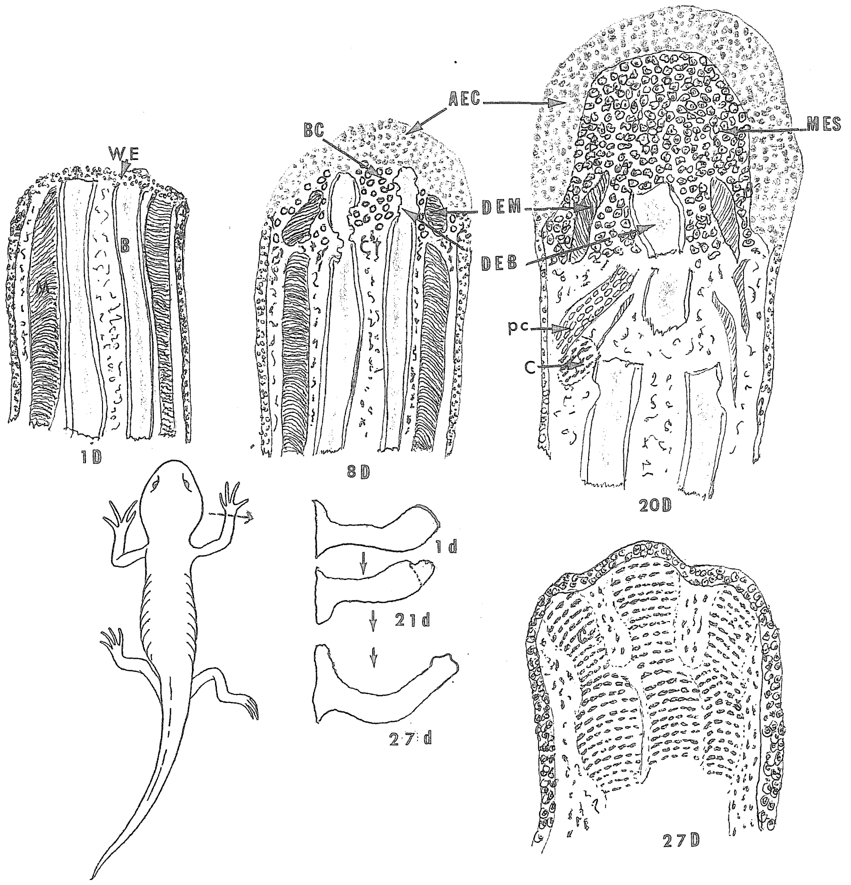

**FIGURE 2.** Adult newt salamander limb regeneration. 1D: 1 day postamputation with wound epithelium; 8D: 8-day regenerate, early blastema with dedifferentiating bone, cartilage, muscle, and apical epidermal cap; 20D: 20-day regenerate (paddle stage) – a collection of mesenchymal cells (blastema), dedifferentiating tissues, and early differentiation of cartilage; 27D: 27-day regenerate (distal end). Early cartilaginous pattern of three toes. M: muscle; B: bone; C: cartilage; AEC: apical epidermal cap; BC: blastema cells; DEM: dedifferentiating muscle; DEB: dedifferentiating bone; MES: mesenchymal cells (blastema); WE: wound epithelium; ct: connective tissue; pc: precartilage.

that after amputation, histolysis of internal tissues occurs. These events happen after close contact of the wound epithelium and underlying tissues[12]. This involves a degradation of the extracellular matrix by acid hydrolases and matrix mettaloproteinases for the release of free cells[13]. Demolition of the damaged cells at the severed inner tissue surfaces occurs by proteolytic enzymes and active phagocytosis of the cell debris[8]. If the epithelial cap is removed, regeneration is blocked[14]. Many others have shown that when the epidermis is absent or when its contact with the internal tissues is blocked, regeneration does not occur[8]. Normally, a basal lamina is not present early at the wound surface in the successful regenerate, but becomes established in the late bud stage[15]. When a basement membrane is formed prematurely under the wound epithelium, regeneration is also blocked[16], and if a full skin flap (epidermis + dermis) covers the stump, the flap prevents blastema formation[17].

Then, the underlying stump cells receive signals, initiated by the activation of thrombin[18,19], which induce changes in the severed tissues of muscle, cartilage, bone, tendons, various types of fibroblasts, and Schwann cells by the process of dedifferentiation[20,21,22,23,24,25,26]. During this phase, differentiated cells with a highly specialized morphological and functional state, e.g., muscle[27], lose their phenotypic identity and apparently return to a progenitor state. In cartilage tissue, nuclei change shape and size as the cartilage matrix disappears, freeing the cells[28]. The syncytial muscle fibers of the amputated stump change into mononucleated mesenchymal cells by dedifferentiation in *Ambystoma* larvae as described with light microscopic studies by Thornton[21,27]. Later, Hay[28] reaffirmed, with meticulous electron microscopic sequences, that muscle tissue dedifferentiated in regenerating *Ambystoma* limbs. The myofibrils first break into pieces (a fragmentation process later described as cellularization)[29] when the individual myofilaments, "Z" bands, and basement membranes are lost. The smooth endoplasmic reticulum within the cells disappears, while the nuclei synthesize DNA. This process transforms the highly differentiated tissues into cell types with "the attributes of the mesenchymal cells from which cartilage and muscle originated in the embryo"[30]. At the same time, resident muscle stem cells are prompted to form progenitor cells within their own lineage[25,31]

As soon as dedifferentiation takes place, cell proliferation follows and mitotic cells can be found in all of the tissues near the amputation surface[32]. The formation of a basal lamina at the wound surface in the late bud stage is essential for continued development. Once formed, it appears to stabilize the phenotype of adjacent cells. The epithelium becomes epidermis and the adjacent mesenchyme becomes dermis[15]. During this period, the wound epidermal cover thickens, changing roughly from three cell layers to 12 or more cell layers to form an "apical epidermal cap" (AEC)[33]. The AEC is believed to emit molecular signals that stimulate and maintain early stages of regeneration[34]. Just beneath the AEC, accumulated mesenchymal cells re-enter the cell cycle, proliferate, and aggregate to form a bud of mitotically active cells called the blastema**.** Using $H^3$ thymidine labeling to trace cell proliferation and migration, Hay and Fischman[35] reported that the blastema cells are derived from dedifferentiating stump tissue. The undifferentiated mesenchymal cells in the proliferating blastema cells synthesize RNA and DNA[36] along with heavy protein synthesis[37], indicating re-entry into the cell cycle. DNA synthesis begins 4–5 days after amputation in tissues interpreted as dedifferentiated muscle, periosteum, cartilage, nerve sheaths, and connective tissues.

The presence of nerves is necessary for the formation of a blastema throughout the early stages of regeneration. This is one of two important differences between embryonic limb formation that does not require nerves for early development; the second is that the embryonic limb bud is vascularized early[8].

Early on, it was found that denervation of limbs would inhibit limb regeneration if performed any time up until 9 days after amputation[38], when nerve dependency ceases. The nerves were postulated to release a trophic factor[30,39] and the presence of nerves was proposed to be a prerequisite for the process of dedifferentiation to take place[12]. Extensive investigations by Singer established that the number or quantity, not the type of nerves, was a controlling factor[40]. Later work by Mescher and Tassava[41] indicated that denervation does not prevent wound healing, histolysis, and dedifferentiation , but failure in the proliferation of mesenchyme cells in the blastema at the amputation surface is the controlling event.

Once a blastema forms, the proliferating mesenchymal cells are directly dependent again on stimuli from the nerves. The neurons release a glial growth factor along with a fibroblast growth factor 2 (FGF2) that initiates cell proliferation[42]. Bead implants with FGF2 into early blastemas of denervated axolotl limbs stimulates regeneration into the digit stages[43]. A third factor is a neurotransmitter, substance P, found in newt ganglia, the AEC, and mesenchyme cells that is mitogenic for blastema cells *in vitro*[44].

Another factor that affects blastema cell proliferation is transferrin, a protein that transports iron and is a cofactor for many enzymes. Transferrin can replace the action of nerve extracts in the stimulation of blastema cell proliferation *in vitro*[45]. It is released from nerves in regenerating axolotl limbs and maintains blastema cells *in vivo*[46]. Mescher proposes that the release of growth factors, including transferrin, from regenerating axons is important for cell proliferation during the avascular phase of blastema growth before such plasma-borne factors are supplied during regeneration. He suggests that transferrin affects the rate of cellular activity[47]. When nerve extracts are immunoabsorpted by antiserum to transferrin, it removes the stimulatory effect on blastema cells. The inhibition can be reversed by the addition of pure transferrin[48].

Other influences controlling blastema cell growth and later dedifferentiation of the organized limb tissues are hormones[49,50], along with several growth factors from the AEC (FGF1, 2, 8, 10). The AEC begins to secrete extracellular matrix proteins, such as laminin and collagen[26]. Hyaluronate synthesis is also stimulated by growing axons[51]. An extensive series of markers for many of these proteins has been identified, along with the identification of homeobox transcription factors, Dlx-3 and Msx-2, and mitogenic signals of the fibroblast growth family[52]. The next phase involves morphogenesis of the limb bud, accompanied by differentiation of the progenitor cells. Redifferentiation of the blastema follows a proximal-distal axis, a sequence that is also found in the limb bud of the developing embryo. The identity of this axis is controlled by a local gene-controlled protein, Prod 1, that is linked to the blastema cell surface[19]. Prod 1 exists as a gradient along the blastema and establishes the positional (proximal-distal) identity of the blastema[19]. The discovery of a ligand for Prod 1, a newt protein (nAG), showed that it plays a key role as a growth factor for blastemal cells and the proximal-distal identity of the blastema[53]. After amputation, nAG appears in the Schwann cells of the regenerating nerves and later in gland cells of the epidermis. The expression of nAG is blocked in the denervated blastema. If the DNA for nAG is introduced by electroporation into the blastema cells of a denervated amputated limb, the expression of nAG returns and the blastema is able to resume the formation of a regenerated limb.

The morphological continuity between the cells of the blastema and redifferentiation of new muscle and cartilage cells is also confirmed at the submicroscopic level. As myoblasts form, the presence of myofilaments and primitive myofibrils are seen along with new protein production[35]. The cells have very large nuclei and nucleoli, and massive amounts of basophilic cytoplasm and ribonucleoprotein are found. The regenerate bud changes its shape to a cone, followed by a pallete configuration. As the progenitor cells begin to differentiate into new cells, they take up positions in the primordium that predict the pattern of the regenerating limb and digit formation. If retinoic acid is applied to a distal limb blastema, it can cause a duplication of proximal limb structures, possibly linked to retinoic acid receptors. It is postulated that the production of retinoic acid activates Hox (homeobox) genes, which dictate pattern formation and proximal-distal positions in the blastema. Signals from the wound epidermis continue to influence further blastema differentiation[52].

## Cell Sources of the Limb Blastema

As noted above, most of the early investigations on the cell origin of the blastema came from histological investigations by light microscopy, electron microscopy, and some transplantation studies. They indicated that mesenchymal cells were the central component of the blastema arising from dedifferentiating stump tissues, muscle, cartilage, nerve sheath cells, dermal fibroblast cells, and, more recently, stem cells[19,26]. Still, the origin of the blastema cells has been controversial The need for establishing the actual lineage of cells from the mesenchymal state with cell tracers and the possible contribution of stem cells, such as muscle satellite cells, has not been completely resolved. At one time, Weiss[54] concluded that the blastema cells arose from undifferentiated reserve cells. Others suggested that the blastema was partly derived from cells of the epidermal cap that were labeled and traced into chondrocytes in the stump area[55]. While radioactive labeling evidence suggests that the epidermis does not contribute cells to the blastema[56], a broader list of cell participants was proposed by Schotte[57] and later others who concluded that all cells and tissues of the stump contributed to the blastema. Basal dermal cells were also proposed as a major source of the mesenchymal cells[58,59]. Most of the stump tissues plus dermal fibroblast cells and Schwann cells, but excluding nerves and blood vessels, appear to be a major source of the limb blastema cells[18,19,23,24,26]. Kintner and Brockes present evidence obtained from antibody screening that Schwann cells are the major source of the blastema cells[60].

Let us look at some of the experimental evidence designed to test the cell origins of the blastema. One study, the effect of dermal cells on the limb patterning process *in situ*, involved the transplantation of labeled skin before limb amputation in the axolotl *A. mexicanum*[59]. Dermal cells are cambial fibroblasts associated with the epidermis. Grafts of limb skin (epidermis and dermis) from genetically marked cells were made to the forelimb before amputation. Skin from a triploid donor was transplanted onto the limb of a diploid host animal after its skin was removed. Later, amputation was made through the grafted skin on the distal half of the arm, exposing the stump tissues of the host (diploid) that were covered with the grafted cuff of triploid skin. Since the epidermis does not contribute to the blastema, the triploid dermal cells were easily identified as they were found throughout the blastema. Cell counts were made based on the number of nucleoli generally found in triploids (3) and diploid tissues (l-2). Histological examinations determined that 19% of all mesodermal tissues were derived from the dermal cells. A previous study on the skin of axolotl indicated that dermal cells are composed of fibroblasts and pigment cells[61] that serve as major reservoirs of cells. Using a cell count analysis on the frequency of triploid cells, Muneoka et al.[59] concluded that dermal cells form 43% of the blastema cell population. This estimate may be too high, since tallying cell numbers by selected tissue sectioning is ambiguous inherent to the technique and the vagaries of recording cells by a nucleolar marker, but the dermal cell contribution to the blastemal cells is well established.

An investigation into the endogenous origins and lineages of the cells in the limb blastema of the axolotl *A. mexicanum,* a return to the earlier experimental probes by Kragl et al., provided a window into the diversity of progenitor cells and a partial answer to their attendant developmental origin[62]. Limb tissues were marked by transgenic procedures using an integrated green fluorescent protein (GFP). Tissue expressing GFP was transplanted to the amputation site of an unlabeled host animal. After amputation, through the engrafted tissues, the fluorescent cells were observed within the blastema and traced into the regenerate. In a second experiment, specific precursor cells within the embryo were labeled, then transplanted into the identical site of nontransgenic embryos. The latter embryos were allowed to develop into juvenile axolotls, their forelimbs amputated, and fluorescent cells traced as the original embryonic precursor cells[63]. As earlier investigations suggested, the results from GFP tracking indicated that the major tissues of the regenerate retain their lineage identity by dedifferentiating to progenitor cells that are lineage restricted. At least four major cell types were found to be lineage restricted, epidermal cells, Schwann cell progeny, muscle, and possibly cartilage.

When GFP+ embryonic presomite mesoderm was transplanted to unmarked host embryos, muscle fibers and satellite cells were labeled (see Fig. 1). After amputation, only GFP+ cells appeared in the 12-day blastema. Those cells that were GFP+ were also positive for the paired box transcription factor (Pax7), a muscle progenitor cell marker, and a myogenic determining factor (Myf3). No GFP+ cells were found in the cartilage or epidermis. Schwann cells were also labeled with GFP+ at the neural fold stage using white mutant embryos as hosts and donors. The GFP+ cells were coincident with neuron-specific βIII-tubulin and a glial cell–specific protein. After amputation and regeneration, GFP+ fluorescence was found only in nerve tracts and in no other cells. No label was found in cartilage or muscle. In addition, labeled cartilage was not found after nerve implants were used to rescue irradiated limbs. In the axolotl, the skeleton consists of cartilage and thus the bone cell lineage was not examined. Instead, pieces of GFP+ upper arm cartilage were transplanted to nonfluorescent hosts before amputation. The GFP+ cartilage cells made major contributions to the blastema population and then regenerated cartilage. No GFP+ cells that were coincident with muscle-derived markers, PAX7 and Myf5, were found in the blastema, nor were GFP+ cells coincident with muscle-specific heavy-chain myosin (MHCI) seen in the regenerated limbs. The labeled GFP+ cells were found in perichondrium, tendons, and possibly dermis.

While the embryonic GFP+ epidermis was restricted only to forming new epidermal cells, the dermal layer provided a large number of blastema cells and evidence indicates their multipotency. Therefore, GFP+ skin was transplanted into nonfluorescent hosts. The labeled tissue included epidermis, Schwann cells, and blood vessels, but excluded muscle cells. The blastema at the mid-bud stage contained a large number of GFP+ dermal cells. There was no overlap of GFP+ and the PAX7+ cells, so muscle progenitor cells were excluded. Also, the polymerase chain reaction (PCR) procedure did not reveal any cells positive for the muscle marker Myf5. In the regenerated limbs, no GFP+ cells were found coincident with PAX+ cells or MHCI+ cells, which further established that dermal cells do not contribute to muscle. Instead, there were significant numbers of GFP+ cells in dermal cells and in new cartilage and tendons.

An experiment for the role of dermis-induced cell progeny was made by labeling GFP+ embryonic lateral plate mesoderm, the primordium for limb dermis, connective tissue, and fibroblasts. Once the animals reached a length of 8 cm, the labeled dermis was transplanted onto nonlabeled host limbs and subsequently the limbs were amputated through the transplant. Cartilage cells developed from the grafted cells, but no GFP+ Schwann cells showed any fluorescence. These results added evidence that the dermal cells contribute to the dermis and form skeletal cartilage and tendons in the regenerate, reinforcing the idea that dermal cells are multipotent stem cells in the axolotl. The potential of the abundant stromal tissue fibroblasts is still not clear and was not determined due to insufficient cell markers. Past evidence[64] suggested that fibroblasts may also be multipotent. These experiments indicated that several of the tissues in the amputated stump, epidermis, muscle, cartilage, and Schwann cells undergo dedifferentiation, but retain their tissue identity, while dermal cells are multipotent stem cells. The fate of interstitial fibroblasts and bone was not tested.

## Contributions to the Blastema from Reserve Satellite Cells

The discovery of satellite cells located just beneath the cell lamina in multinucleated skeletal muscle cells in mammals[65] led to the proposal that they are true, specific reserve, stem cells and that they are involved in repair of injured muscle[25]. Muscle stem cells have been implicated in the growth, repair, regeneration, and muscle stem cell therapy in higher vertebrates and mammals[1]. In urodeles, they are unique in that they are completely surrounded by their own external lamina and exist separately from the myotubes. They can transform into myoblasts, fuse with resident myofibers, contribute to normal growth of muscle, and regenerate muscle following injury[66].

Indirect evidence suggested that satellite cells could regenerate myotubes *in vitro* and might be involved in the regeneration of muscle from the blastema of the adult newt[67]. The stem cells could incorporate $H^3$ thymidine and proliferate, then fuse into myotubes and express both blastema and myoblast specific antigens.

Related studies on tadpole tail regeneration in *Xenopus* suggested that muscle may be derived from satellite cells. In this system, there is no dedifferentiation of multinucleated muscle cells. It was also shown that the specific transcription factor (Pax7) may play a role in satellite cell regeneration of muscle in the tadpole, since inhibition of Pax7 prevented muscle regeneration[67].

A recent test was applied to determine if dedifferentiation also triggers activation of resident muscle stem cells and, if so, whether these cells contributed to the new limb[31]. Satellite cells in the newt muscle were identified with an antibody against Pax7. At 4 days after amputation, histological examinations indicated that the satellite cells were activated by the presence of a mitotic marker, histone 3 (CH3P), and detected by Pax7+ cells in the blastema. Next, a cell culture was established from single newt myofibers that were accompanied with associated satellite cells. The syncytial cells of the myofibers were injected with a fluorescein-conjugated nuclear-localizing dexstran (NLS-dexstran) that was excluded by the satellite nuclei, while the satellite cells remained Pax7+. After induced cellularization *in vitro*, the mononucleated cells contained unlabeled nuclei that had entered the S phase and could be labeled with bromodeoxyuridine (BrdU), a marker for DNA synthesis. The satellite cells migrated out, retained Pax7 expression, and were positive for the muscle cell marker MyoD. When the latter were cultured in a myogenic medium, they formed myotubes. In addition, under appropriate conditions, the stem cell progeny were found to be multipotent since they had both adipogenic and osteogenic potential. In further experiments, BrdU-labeled satellite cell progeny were injected intramuscularly before amputation. At the medium limb bud stage, the labeled cells were located within the blastema and, surprisingly, in the epidermis. In the late regenerating bud, labeled cells were also found in the newly formed cartilage. These results suggested that satellite cells make a significant contribution to the blastema and limb regenerate[69].

Satellite cell activation occurs *in situ* in response to limb amputation and later labeled cells appear in the early bud blastema. Since resident myofibers are also dedifferentiating, this is not surprising. While the implication that satellite cells contribute to new muscle from the limb blastema regenerate is quite likely and plausible, their relative role in the regenerating limb bud is still arguable. The bulk of these and previous experiments on satellite cells results from their developmental behavior in culture and may not measure their actual role *in vivo*. Other caveats are raised by Slack[66] about the possible contributions of nonmuscle cells associated with the myoblasts in culture. Such side populations include hematopoietic stem cells and Schwann cells, both of which exhibit metaplasia. The most important source would be connective tissue fibroblasts from the sheath and fascicles of muscle fibers that could undergo metaplasia. Thus, the relative contribution of myofibers and satellite cells is still not clear. A simultaneous cell lineage tracing *in vivo* both satellite cells and myoblasts into definitive tissues might help to resolve this issue.

## Inherent Potential of Blastema Cells

Attendant to the ultimate cell source of the blastema is the developmental potential of the blastema cells. Are blastema cells equivalent to embryonic mesenchyme cells in their potency? The progenitor blastema cells, while not identical to embryonic primordial cells, may be precursor cells that are capable of redifferentiating into all specialized tissues, muscle, bone, cartilage, fibroblasts, etc., somewhat like limb development from embryonic mesenchyme. The inherent potential of blastema cells has been raised before[11,12]. Do these cells “know” where they came from and thus redifferentiate only into their own cell type? Even so, can they transform (transdifferentiate) into other closely related cell types that are normally found in the regenerate? Alternatively, some cells might be multipotent stem cells and capable of forming more than one cell type of the regenerating limb. Different models for blastema cell plasticity propose unipotent (restricted) cells, multipotent cells, or a mixture of restricted and multipotent cells[68]. In contrast to the developing limb bud in the embryo, where forelimb or hindlimb buds can be exchanged and either can adapt to the new region after transplantation, the limb blastema does not have morphogenetic plasticity, i.e., the forelimb blastema cannot adapt to the hindlimb site or tail region when transplanted ectopically[69]. Thus, a concept has developed that the multipotent cells of the blastema are equivalent to multipotent stem cells found in adult tissues[26,69]. Indeed, it has been proposed that dedifferentiated cells in salamander regenerates and mammalian stem cells share the same molecular signatures[70]. A comparison of the mammalian transcriptional factors used to induce iPS cells[2] has been made with similar expression in the regenerating iris and newt limb blastema[71]. Three of these factors, Sox2, Klf4, and the oncogene c-myc, were expressed in newt blastema cells. Two other factors, Oct 4 and Nanog, were not found.

## The Blastema “Field Effect”

Certain zones of the normal developing embryo, called embryonic fields, can be identified as groups of precursors cells for specialized organs or structures, where cell differentiation and pattern formation are progressively localized that are inherent to the nature of the organ being formed. The limb primordium is one example of an embryonic field. Differentiation and pattern formation of the limb regenerate are also a field characteristic of the blastema[7]. The limb development field can be recognized by several characteristics, such as its organizational and regulatory capability. The limb regeneration blastema also shares this characteristic and can regulate either for the loss of cells from the early blastema or the addition of a limb blastema to form a single limb. When undifferentiated early embryonic cells from another organized area or blastema are transplanted into the limb field, they are assimilated into the limb field and respond to it by contributing to the normal developing limb.

If blastema cells from the sectioned intestine of the adult newt are labeled with radioactive thymidine (either *in vitro* or *in vivo*) and placed into a limb blastema just before redifferentiation into skeletal tissues, the implanted cells are incorporated into the limb blastema. The tagged cells now show up in newly formed cartilage[72]. These experiments, if substantiated, would suggest that the intestine blastema cells, which never form cartilage in their original site, could be transformed into cartilage precursors by the influence of the resident limb blastema.

Furthermore, the blastema field can cause differentiated cells to dedifferentiate. When differentiated chrondrocytes from the axolotl limb of a triploid animal are labeled *in vitro* with $H^3$ thymidine and then implanted into a diploid limb blastema below the epidermal cap, they become mononucleated and are incorporated into blastema cells. After differentiation, they retain their lineage and, ultimately, the labeled cells are found in the cartilage and in connective tissue of the regenerate[73]. Yet other labeled muscle cells redifferentiated into myoblasts, but some also formed chondrocytes during regeneration; however, the exact origin of the muscle cell types was not identified, i.e., muscle, connective tissue, or endothelial cells. Thus, this experiment needs to be repeated. The blastema has been recognized as an environment that destabilizes the differentiated state of cells[24], returning them to a progenitor state. Thus, it appears that the blastema is able to assimilate and instruct progenitor cells and certain differentiated cells. As a result, various studies have focused on identifying cells with markers and then transplanting them into the blastema in order to determine their possible contribution to the regenerate. In one study, differentiated cells from the triploid larval axolotl limb were grafted into the diploid X-irradiated limbs of host animals[74]. If the control irradiated limbs that had not received grafts were amputated, they could not regenerate. After implantation of labeled cartilage cells into the limb followed by amputation, the cartilage cells dedifferentiated and later redifferentiated into triploid-derived cartilage, perichondrium, connective tissue, and fibroblasts. Cell types derived from muscle also dedifferentiated and later differentiated into mesodermal tissues of the limb in addition to cartilage. Two other interpretations for these apparent events of transdifferentiation are possible: (1) the presence of multipotent (stem) cells that could then differentiate into mesenchymal-derived cell types or (2) dedifferentiation of the cartilage cells into progenitor cells that are already committed to different lineages[58] .

Likewise, if blastema cells of the newt *Notophthalmus* are isolated and grown in tissue culture, some of the cells will fuse to form myotubes when the serum levels are lowered[75]. These differentiated multinucleated myotubes were labeled with a lineage tracer of rhodamine-conjugated dextran in the cytoplasm and microinjected into the blastema 3–7 days after amputation[76]. In parallel experiments, a second label with $H^3$ thymidine was also applied to the microtubules. When the labeled myotubes were transplanted back into the blastema, the precursor multinucleated myotubes reversed differentiation, yielding strongly labeled mononucleated cells that continued to proliferate. After 1 week, myoblast cells were observed whose numbers multiplied with time and were found almost exclusively in undifferentiated mesenchyme of the blastema. Cell labeling allowed them to be traced. Dedifferentiation was completed at the mid-cone stage (2–3 weeks after amputation) and cells were widely distributed through the host mesenchyme. These results indicated that the implanted myotubes dedifferentiated within the environment of the “limb field” blastema. Some of the injected cells appeared to fuse with existing muscle fibers and, later, some of the labeled cells were found in pods of newly regenerated cartilage, suggesting transdifferentiation. Yet, they could have resulted from cell fusion with predetermined cartilage progenitors, a problem often encountered when tracing the progeny of adult tissue stem cells. These experiments also suggest that the microenvironment of the blastema field can reverse the differentiation of specialized cells and return them to the progenitor state. Further evidence was obtained when radioactively labeled blastemal cells from the regenerating tail inserted into the 14-day limb blastema formed regular limb cartilage.

Kumar et al.[77] extended the previous work on myotube dedifferentiation when they used cultured newt A1 myotubes to probe the mechanisms of plasticity in the blastema. The indices used were changes in mononucleated muscle cells on fusion into myotube and postmitotic arrest. Concomitantly, the tubes are refractory to growth factors that stimulate the division of mononuclear myoblasts. The cells also expressed several markers of muscle differentiation. First, the investigators infected A1 mononucleated muscle cells *in vitro* with a genetic marker, a pseudotyped retrovirus that expressed human placental alkaline phosphatase. Separately, they labeled A1 cells with a fluorescent lipophilic cell (membrane) marker. Another approach utilized a tracker fluorochrome and a nuclear dye (Hoeschst) for DNA. After fusion, myotubes were microinjected under the epidermis of forelimb blastemas at 4–5 days after amputation. The limb blastemas were harvested 10 days later. In all three procedures, labeled mononucleated cells were found in the blastema and in the stump tissues near the amputation plane. After implantation of the retroviral tagged myotubes, they were injected with BrdU, both *in vitro* and *in vivo*, 9 days after implantation. Labeled nuclei were high (range: 42–86%), which indicated that the myotubes had entered the S phase of the cell cycle in the blastema microenvironment. These results showed that the blastema causes the labeled cells to dedifferentiate and contribute cells to the blastema, thus partially confirming the previous results of Lo and associates[76], but they did not confirm conversion of myotubes to muscle or cartilage.

## Transdifferentiation

Cell transformation or transdifferentiation has been defined as the direct conversion of one differentiated cell type into another differentiated cell type[78]. A sterling example of transdifferentiation in adult mice is the recent discovery by Zhou et al.[79], who induced transdifferentiation of exocrine cells into β-islet cells of the pancreas using four main transcription factors needed for dedifferentiation. As such, transdifferentiation has been proposed as the change of a highly specialized cell into another specialized cell circumventing dedifferentiation to a progenitor state[80].

Another example of possible cell transformation is the regeneration of the eye lens in newts, which has been investigated by many for years[7]. After removal of the eye lens, the differentiated cells of the dorsal pigmented iris can dedifferentiate and then reform into lens cells. The pigmented epithelial cells (PECs) lose their pigment, form a vesicle, and then redifferentiate into new specialized cells of the lens and lens epithelium. A review of this transformation in the eye chamber by Tsonis and Del Rio-Tsonis[81] has proposed it to be transdifferentiation. Therein, they propose that transdifferentation is a process by which terminally differentiated cells are able to dedifferentiate (loss of characteristics of their origin) and subsequently redifferentiate. Significantly, the PECs of the dorsal iris from the adult newt that dedifferentiate to form nonpigmented precursors before differentiating into lens will not transdifferentiate when transplanted anywhere in the body except when they are placed into the limb blastema environment[82].

Associated evidence from urodeles for transdifferentiation of cells between germ layers that involves stem cells is seen in the blastema of the regenerating axolotl tail. After tail amputation, dedifferentiation of muscle, fibroblasts, and cartilage occurs to populate the blastema. Additionally, the spinal cord has abundant neural stem cells (NSCs) that form neural precursor cells that are released from the spinal cord into the blastema. The neural tube regenerates the spinal cord and neural crest derivatives. There is indication that some of the NSCs transdifferentiate into muscle cells and chondrocytes of cartilage[83]. After tail amputation, cDNA for GFP plus a promoter, glial fibrillary acidic protein (GFAP), specific to ependymal cells of the mature spinal cord, were inserted into spinal cord ependymal cells. It was possible to track ependymal cells that were verified by antibodies to the GFAP promoter, into neurons and glia of the spinal cord and neural crest derivatives. Some cells left the regenerating ependymal tube and transdifferentiated into muscle (24%) and precartilage chondrocytes (12%). The muscle cells were identified by antibodies to heavy-chain myosin specific for muscle. Two explanations for these events were offered by Stocum[84]. It is possible that the NSCs fuse with blastema cells already programmed to form muscle. Second, the NSCs might respond to signals from the blastema (field effect), causing transdifferentiation into myoblasts that later fuse into muscle myotubes.

While there is some evidence of transdifferentiation of exogenous cells introduced into the limb blastema, it has not been proven that mesenchyme progenitor cells of the blastema undergo transdifferentiation, i.e., direct conversion of one specialized cell into another. If some of the resident blastema cells *in situ* are shown to transform into two or more cell types, this could also result from the formation of multipotent stem cell progenitor cells. When previous myotubes dedifferentiate into blastema cells, retain their lineage, and redifferentiate into new muscle cells as the present histological and transplantation data indicate, this supports dedifferentiation. The essential difference is that the blastema cells are composed of progenitor cells derived from muscle, cartilage, fibroblasts, etc. that proliferate, differentiate, and reorganize to form a complex amphibian limb[19]. The latest results on *in*

*situ* cell tracing[62] indicate that many of the cell types maintain their cell lineage after dedifferentiation, along with progeny from multipotent dermal stem cells and possibly resident reserve muscle cells.

## Parameters Regulating Progenitor Cells

Cell differentiation of embryonic limb primordia, adult stem cells from diverse tissue, and limb blastema cells all share similar molecular pathways of growth and differentiation. Not surprisingly, studies on adult stem cells in mammals indicated that the control of progenitor cell growth and differentiation resulted from the interaction of several ubiquitous growth factors and cytokines, EGF, bFGF, NGF, TGF-β (transforming growth factor), which trigger stem cell growth and differentiation[1].

The regulation of embryonic and adult stem cells and embryonic-like pluripotent stem cells during differentiation is mediated by cell-to-cell interactions and proteins of the extracellular matrix via signal transduction pathways that activate gene-controlled transcription factors[2]. Some typical pathways, in addition to the tyrosine kinase receptor pathway, are the Notch and Wnt pathways involved in stem cell maintenance and progenitor cell proliferation, the Hedgehog pathway, the TGF-β pathway (regulates growth and differentiation), and a janus kinase signal transducer activator of transcription (JAK-Stat) pathway necessary for somatic stem cell maintenance. An excellent synopsis of the six major signaling pathways demonstrated during embryonic development and in adult stem cells is found in Stocum[85].

The effect of growth factors and other proliferation stimuli were tested on newt A1 myotubes described earlier in experiments involving implantation of newt A1 cells into the limb blastema. The newt myotubes also responded to EGF and FGF, along with platelet-derived growth factor (PDGF) and insulin-like growth factor (IGF)[19]

Several pathways have been identified as controlling the process of dedifferentiation of the blastema. The Wnt signaling cascade regulates the expression of specific targeted genes and determines cell fate decisions and morphogenesis. A key to the Wnt pathway is the effector molecules β-catenin and a destruction complex that controls its stability. In normal limb development and early limb bud formation, Wnt glycoproteins elicit signals through the "canonical" β-catenin pathway[86,87,88]. Two noncanonical genes, Wnt-5a and Wnt-5b, are also expressed and regulate dedifferentiation in the axolotl limb blastema[89]. When Wnt-5a is linked to a vaccinia-based vector, it is expressed in different regions and in early, medium, and late blastemas. Injections at the junction between the amputation stump and the base of the blastema inhibits the effect of Msx-2 and thus appears to regulate dedifferentiation, producing a hypomorphic limb. Based on expression patterns, it was hypothesized that ectopic Wnt-5a expression inhibits canonical Wnt signaling.

A number of myogenic genes have been implicated in muscle differentiation and dedifferentiation in developing and regenerating axolotl limbs. These include Msx-1 (Hox7) a muscle homeobox gene that is also found in the mouse and human. Msx-1 is believed to maintain proliferation of undifferentiated cells in the embryonic salamander limb bud and is up-regulated at the initiation of salamander limb regeneration[90]. Msx-1 produces a protein that acts as a transcriptional repressor during embryogenesis. Expression of Msx-1 is involved in the induction of dedifferentiation of multinuclear mouse myotubes by causing them to cleave via cellularization into mononucleated muscle progenitor cells. Two other myogenic regulatory factors are MRF-4 that is active in adult muscle, but is turned off in the early regeneration blastema, and a second is Myf5 that is expressed throughout limb regeneration. The derived muscle progenitor cells were multipotent since they could redifferentiate into cells having markers for multiple cell types, myotubes, cartilage, fat, and bone[29]. These regulatory factors may function by controlling cell plasticity of muscle progenitor cells (myoblasts) and may induce dedifferentiation into multipotent progenitor cells. To test these projected functions, muscle fibers from the limbs of larval salamanders (*A. maculatum* and the axolotl *A. mexicanum)* were isolated and placed in culture[91]. Single fibers were isolated and injected with Cell Tracker Orange and a nuclear dye, Sytox green. Adherent mononucleated satellite cells were stained by a nuclear dye and so any adherent satellite cells were removed from the cultures before cellularization of the myofibers. The lineage-tagged myofibers, devoid of mononucleated cells, were then injected into forelimb blastemas 48 h after amputation. Two to four days later, the implanted cells' myofibers had produced mononucleated progeny identified by the tracer dyes, and they also exhibited Msx-1–derived mRNA and protein. In separate experiments, the use of antisense oligonucleotides decreased the Msx-1 protein in the myoblasts and inhibited cellularization. The conclusions were that Msx-1 is necessary for initiating the events leading to cellularization that result in plasticity of the mononucleated muscle cells.

Formerly, several Hox genes were identified as being expressed in both developing and regenerating axolotl limbs[91,92]. Another family of homeobox genes was isolated from the newt that controls forelimb development as it is formed from flank mesoderm[93]. These genes possess a novel DNA-binding domain, a conserved T-box shared with the Brachyury (T) gene in the mouse. Using a mRNA differential display for cDNA tags, a family of T-box genes was isolated in the newt. The newt gene (NvTbox1) activity is elevated in the mesenchyme of the undifferentiated limb bud during embryogenesis. The activity of the same gene is also expressed in the progenitor cells in the proximal blastema of the larval forelimb when it is stimulated by retinoic acid. The NvTbox1 gene was expressed uniformly throughout the limb mesenchyme in both the developing forelimb bud and the mid-bud blastema. Thereafter, the activity is diminished in the early digital stage and fully regenerated forelimb. This supports the hypothesis that the T-box gene is influential in axial positional information both in the developing new limb and in the regeneration blastema.

There is a possible link between stem cell proliferation, limb regeneration, and tumor cell suppression in a related species. Earlier work showed that the protein PTEN (phosphatase and tensin homologue) acts as a tumor suppressor of proliferation and possibly restores pluripotency in cells. A deficiency of PTEN increases neural stem cells and their progenitor cells[1]. Another embryonic stem cell factor is SALL4, which plays a role in both embryonic and leukemic stem cells. This stem cell factor represses the transcriptions of genes for both PTEN and SALL4[94]. A conserved stem cell factor involved in both development and regeneration of the fore- and hindlimb regeneration in *X. laevis* is XlSALL4, a member of the SAL family. Transcripts for XlSALL4 have been found during normal fore- and hindlimb development and are expressed along the anterior/posterior axis during limb development, and have the potential for regulating epimorphic regeneration[95].

## SUMMARY AND DISCUSSION

### Limb Blastema Cell Origins

In limb regeneration, specialized cells at the injury site, in response to local signals, begin to dedifferentiate, then proliferate to form a blastema of mesenchymal cells with mixed developmental potential. These cells then redifferentiate back to their original lineage or they may transdifferentiate into new cell types[19]. The cellular origin of the limb regenerate blastema appears to be a mixture of dedifferentiated cells or adult stem cells[85]. Differentiated tissues derived from bone, cartilage, muscle, fibroblasts, and Schwann cells dedifferentiate and are lineage restricted to their tissues of origin as demonstrated by Hay[30] and Kragl et al.[62]. Dermis-derived fibroblasts act as multipotent stem cells producing progenitor cells for cartilage, connective tissues, and tendons[62]. The cellular contributions of other connective tissue fibroblasts have been hampered by the lack of cell markers, but based on dermal cell contributions, they are probably heavily involved. The best evidence for the contributions of muscle satellite cells is the activation of multipotent Pax7+ satellite cells in limb regenerates[69]. It has not been established how much they contribute to the new regenerate and further lineage studies are needed. The model depicting the limb blastema cells as a collection of uniformly pluripotent cells has been negated and most evidence indicates that the blastema is composed of a mixture of progenitor cells and stem cells with several origins and capabilities. The progression of dermal stem cells into fibroblasts and progenitor cells of cartilage needs further investigation, along with a determination of the contribution that stromal fibroblasts may play during blastema differentiation.

## Epigenetics

An interesting parallel between the levels of developmental potential and their epigenetic status has been presented by Hochedlinger and Plath[4]. Other than the totipotency of the zygote, pluripotent stem cells, including embryonic cells, cell lines from germ cells, carcinoma cell lines, and iPS cells, all express active X chromosomes, repression of differentiation genes, and promoter hypomethylation. Multipotent adult stem cells exhibit X-chromosome inactivation, repression of lineage-specific genes, and promoter hypermethylation. Differentiated cell types (unipotent) exhibit X-chromosome inactivation, derepression of polycomb-silenced genes, and promoter hypermethylation. Hochedlinger and Plath conclude that restrictions on the genome during development must be due to reversible epigenetic changes. Thus, cells in different levels of differentiation can be reprogrammed by transcription factors back to different levels of potential, pluripotent, multipotent, or specified progenitor cells. As this profile is augmented, the role of these and other factors may be exploited in order to understand differentiation, stem cells, and progenitor activity in the regeneration blastema.

## The Blastema Field

The blastema field of urodeles has the capability to regulate for the random loss of blastema cells, accommodate the introduction of new blastema cells, and form multiple regenerates from a single blastema field[11]. It can also destabilize the differentiated state[17], perhaps forming progenitor cells for related cell types. The importation of differentiated exogenous cells into the limb blastema that are incorporated in the regenerating limb is often cited as evidence for the cellular origin of the blastema; however, it is not established whether the same cells are contributors to the *in situ* blastema. Attempts to utilize the results from limb and other regenerating systems in mammalian regenerative medicine reflects two general experimental approaches[18,58]. The first emphasizes the introduction of exogenous progenitor cells or differentiated cells into the blastema[11], and the second suggests activation of resident stem cells or progenitor cells at the amputation site[58]. The implantation of progenitor cells or differentiated cells into the blastema enabled their progeny to be traced into new cell types within the regenerate, an expansion of their developmental potential. When differentiated myotubes are implanted into a blastema and reverse their differentiation, it was interpreted as the plasticity of the differentiated state and a reversal of their differentiation[53]. Just as stem cells are often dependant on their niche, the blastema enables cells to dedifferentiate to the progenitor state. Similar to this is the process of SCNT (see Introduction) in mammals. Implicit in the technique is that the host cell provides a microenvironment that permits the donor nucleus to be reprogrammed into a pluripotent state, a process that is poorly understood. As these factors are identified, they may lead to identification of similar factors that operate in the generation of progenitor cells in the blastema. An interesting question about comparative regenerative abilities between larval and adult forms has been raised by Gurlely and Alvarado[58], i.e., what are the epigenetic differences between adult and larval axolotls? It is possible that the epigenetic profiles could vary between these two developmental stages, in addition to intraspecies differences.

The phenomenon of transdifferentiation, exemplified by the transformation of neural stem cells into muscle and cartilage in the tail blastema is well documented, but the process is still subject to interpretation as to the extent of dedifferentiation involved. Experiments on the introduction of exogenous cells into the blastema provide important information on their transdifferentiation potential. The direct conversion of a differentiated cell into another differentiated cell, as in lens regeneration or in the conversion of pancreatic cells, has been described as lineage conversion or transdifferentiation caused by an ectopic deletion or addition of a transcription factor[4]. As such, it may not involve a gain in differentiation potential. It has not been established whether this form of reprogramming involves dedifferentiation back to the progenitor state, as takes place in the reprogramming of adult cells into iPS cells. Perhaps it would be more significant if the focus was on what in the blastema “field” constitutes the regenerative signals that induce dedifferentiation or the putative transdifferentiation.

## ACKNOWLEDGMENT

The author would like to thank the Editor of Developmental and Embryology of *TheScientificWorldJOURNAL* and the staff of the Marine Biological Laboratory-Oceanographic Institution Library in Woods Hole, MA.

## References

1. Tweedell, K.S. (2004) Embryos, clones and stem cells. *TheScientificWorldJOURNAL* **4**, 662–715.
2. Tweedell, K.S. (2008) New paths to pluripotent stem cells. *Curr. Stem Cell Res. Ther.* **3,** 151–162.
3. Takahashi, K. and Yamanaka, S. (2006) Induction of pluripotent stem cells from mouse embryonic and adult fibroblast cultures by defined factors. *Cell* **126** 663–676.
4. Hochedlinger, K. and Plath, K. (2009) Epigenetic reprogramming and induced pluripotency. *Development* **136,** 509–523.
5. Balinsky, B.I. (1981) *An Introduction to Embryology.* 5th ed. Saunders College, Philadelphia.
6. Harrison, R.G. (1918) Experiments on the development of the forelimb of *Amblystoma,* a self-differentiating equipotential system. *J. Exp. Zool.* **25,** 413–461.
7. Gilbert, S. (2006) *Developmental Biology*. 8th ed. Sinauer Associates, Sunderland, MA.
8. Tank, P.W., Carlson, B.M., and Connelly, T.G. (1976) A staging system for forelimb regeneration in the axolotl *Ambystoma mexicanum. J. Morphol.* **150,** 117–128.
9. Carlson, B.M. (1998) Development and regeneration, with special emphasis on the amphibian limb. In *Cellular and Molecular Basis of Regeneration.* Ferretti, P. and Geraudie, J., Eds. John Wiley & Sons, New York.
10. Stocum, D.L. and Fallon, J.F. (1982) Control of pattern formation in urodele limb ontogeny. *J. Embryol. Exp. Morphol.* **69,** 7–36.
11. Goss, R.J. (1969) *Principles of Regeneration*. Academic Press, New York.
12. Rose, S.M. (1948) The role of nerves in amphibian limb regeneration. *Ann. N. Y. Acad. Sci.* **49**, 818–833.
13. Yang, E.V., Gardiner, D.M., and Bryant, S.V. (1999) Expression of Mmp-9 and related matrix metalloproteinase genes during axolotl lib regeneration. *Dev. Dyn.* **216,** 2–9.
14. Thornton, C.S. (1957) The effect of apical cap removal on limb regeneration in *Amblystoma* larvae. *J. Exp. Zool.* **134,** 357–381.
15. Neufeld, D.A. and Day, F.A. (1998) Wound epidermis perspective: a suggested role for basement membrane structures during newt regeneration. *Anat. Rec.* **246,** 155–165.
16. Stocum, D.L. and Crawford, K. (1987) Use of retinoids to analyze the cellular basis of positional memory in regenerating axolotl limb. *Biochem. Cell Biol.* **65**, 750–761.
17. Mescher, A.L. (1976) Effects on adult newt limb regeneration of partial and complete skin flaps over the amputation surface. *J. Exp. Zool.* **195,** 117–128.
18. Brockes, J.P. and Kumar, A. (2005) Appendage regeneration in adult vertebrates and implications for regenerative medicine. *Science* **310**, 1919–1923.
19. Straube, W.L. and Tanaka, E.M. (2006) Reversibility of the differentiated state: regeneration in amphibians. *Artif. Organs* **30(10),** 743–755.
20. Butler, E.G. (1933) Studies on limb regeneration in x-rayed *Ambystoma* larvae. *Anat. Rec.* **62,** 295–307.
21. Thornton, C.S. (1938) The histogenesis of muscle in the regenerating forelimb of larval *Amblystoma punctatum. J. Morphol.* **62**, 17–47.
22. Hay, E. (1958) The fine structure of blastema cells and differentiating cartilage cells in regenerating limbs of *Amblystoma* larvae. *J. Biophys. Biochem. Cytol.* **4**, 583–592.
23. Mescher, A.L. (1996) The cellular basis of limb regeneration in urodeles. *Int. J. Dev. Biol.* **40,** 785–795.
24. Brockes, J.P. (1998) Progenitor cells for regeneration: origin by reversal of the differentiated state. In *Cellular and Molecular Basis of Regeneration.* Ferretti, P. and Geraude, J., Eds. John Wiley & Sons, New York.
25. Odelberg, S.J. (2002) Inducing cellular dedifferentiation: a potential method for enhancing endogenous regeneration in mammals. *Semin. Cell Dev. Biol.* **13**, 335–343.
26. Stocum, D.L. (2006) Regeneration of appendages. In *Regenerative Biology and Medicine.* Elsevier, Burlington, MA. Chap. 14.
27. Thornton, C.R. (1942) Studies on the origin of the regeneration blastema in *Triturus viridescens. J. Exp. Zool.* **89**, 375–390.
28. Hay, E. (1959) Electron microscopic observations of muscle dedifferentiation in regenerating *Amblystoma* limbs. *Dev. Biol.* **1**, 555–583.
29. Odelberg, S.J., Kollhoff, A., and Keating, M.T. (2000) Dedifferentiation of mammalian myotubes induced by msx 1. *Cell* **103**, 1099–1109.

30. Hay, E. (1962) Cytological studies of dedifferentiation and differentiation in regenerating amphibian limbs. In *Regeneration.* Rudnick, D. Ed., Ronald Press, New York. pp.177–210.
31. Morrison, J.I., Loof, S., He, P., and Simon, A. (2006) Salamander limb regeneration involves the activation of a multipotent skeletal muscle satellite cell population. *J. Cell Biol.* **172,** 443–440.
32. Chalkley, D.T. (1954) A quantitative histological analysis of forelimb regeneration in *Triturus viridescens. J. Morphol.* **94**, 21–70.
33. Singer, M. (1959) The influence of nerves on regeneration. In *Regeneration in Vertebrates.* Thornton, C.S., Ed. University of Chicago Press. pp. 59–80.
34. Whited, J.L. and Tabin, C.J. (2009) Limb regeneration revisited. *J. Biol.* **8,** 5.
35. Hay, E. and Fischman, D.A. (1961) Origin of the blastema in the regenerating newt *Triturus viridescens.* An autoradiographic study using tritiated thymidine to follow cell proliferation and migration. *Dev. Biol*. **3,** 26–59.
36. Hay, E. (1965) Metabolic patterns in limb development and regeneration. In *Organogenesis.* Detlaan, R.L. and Ursprung, H., Eds. Holt, Rinehart & Winston, New York. pp. 314–356.
37. Anton, H.J. (1965) The origin of blastema cells and protein synthesis during forelimb regeneration in *Triturus.* In *Regeneration in Animals and Related Problems.* Kiortsis, V. and Trampusch, H.A.L., Eds. North Holland, Amsterdam.
38. Schotte, O.E. and Butler, E.G. (1944) Phases in regeneration of the urodele limb and their dependence upon the nervous system. *J. Exp. Zool.* **97,** 95–121.
39. Thornton, C.S. (1970) Amphibian limb regeneration and its relation to nerves. *Am. Zool.* **10,** 113–118.
40. Singer, M. (1978) On the nature of the neurotrophic phenomenon in urodele limb regeneration. *Am. Zool.* **18,** 829–841.
41. Mescher, A.L. and Tassava, R.A. (1975) Denervation effects on DNA replication and mitosis during the initiation of limb regeneration in adult newts. *Dev. Biol.* **44,** 187–197.
42. Brockes, J.P. (1987) The nerve dependence of amphibian nerve regeneration. *J. Exp. Biol.* **132,** 79–91.
43. Mullen, L.M., Bryant, S.V., Torok, M.A., Blumberg, B., and Gardiner, D.M. (1996). Nerve dependency of regeneration: the role of distal-less and FGF signaling in amphibian limb regeneration. *Development* **122,** 3487–3497.
44. Globus, M. and Alles, P. (1990) A search for immunoreactive substance P and other neural peptides in the limb regenerate of the newt, *Notophthalmus viridescens. J. Exp. Zool.* **254,** 165–176.
45. Munaim, S.I. and Mescher, A.L. (1986) Transferrin and the trophic effect of neural tissue on amphibian limb regeneration blastemas. *Dev. Biol.* **152,** 138–142.
46. Mescher, A.L. and Kiffmeyer, W.R. (1992) Axonal release of transferrin in peripheral nerves of axolotls during regeneration. In *Keys for Regeneration*: *Monographs in Developmental Biology.* Vol. 23. Taban, C.H. and Boilly, B., Eds. Karger, Basel. pp. 100–109.
47. Mescher, A.L. (1996) The cellular basis of limb regeneration in urodeles. *Int. J. Dev. Biol.* **40,** 785–795.
48. Mescher, A.L., Connell, E., Hsu, C., Patel, C., and Overton, B. (1997) Transferrin is necessary and sufficient for the neural effect on growth in amphibians limb regeneration blastema. *Dev. Growth Differ.* **39,** 677–684.
49. Liversage, R.A., McLaughlin, D.S., and McLaughlin, H.M.G. (1985) The hormonal milieu in amphibian appendage regeneration. In *Regulation of Vertebrate Limb Regeneration*. Sicard, R.E., Ed. Oxford University Press, New York. pp. 54–80.
50. Tsonis, P.A. (1996) *Regeneration*. Cambridge University Press, Cambridge.
51. Mescher, A.L. and Cox, C.A. (1988) Hyaluronate accumulation and nerve dependent growth during regeneration of larval *Ambystoma* limbs. *Differentiation* **38,** 161–168.
52. Campbell, L.J. and Crews, C.M. (2008) Wound epidermis formation and function in urodele amphibian limb regeneration. *Cell. Mol. Life Sci.* **65.,** 73–80.
53. Kumar, A., Godwin, J.W., Gates, P.B., Garzza-Garcia, A.A., and Brockes, J.P. (2000) Molecular basis for the nerve dependence of limb regeneration in an adult vertebrate. *Science* **318,** 772–777.
54. Weiss, P. (1939) *Principles of Development.* Henry Holt, New York.
55. Rose, S.M. and Rose, F.C. (1965) The role of normal epidermis in recovery of regenerative ability in x-ray limbs of *Triturus. Growth* **29,** 361–393.
56. Riddiford, L. (1960) Autoradiographic studies on tritiated thymidine infused into the blastema of the early regenerate in the adult newt, *Triturus. J. Exp. Zool.* **144,** 25–32.
57. Schotte, O. (1939) The origin and morphogenetic potencies of regenerates. *Growth* **3(Suppl.)** 59–76.
58. Gurley, K.A. and Alvarado, A.S. (2009) Stem cells in animal models of regeneration. In *StemBook.* http://www.stembook.org/node/533
59. Muneoka, K., Fox, W.F., and Bryant, S.V. (1986) Cellular contribution from dermis and cartilage to the regenerating limb blastema in axolotls. *Dev. Biol.,* **116,** 256–260.
60. Kintner, C.R. and Brockes, J.P. (1985) Monoclonal antibodies to the cells of regenerating limb. *J. Embryol. Exp. Morphol.* **89,** 37–55.
61. Holder, N. and Glade, R. (1984) Skin glands in the axolotl: the creation and maintenance of a spacing pattern. *J. Embryol. Exp. Morphol.* **79,** 97–112.
62. Kragl, M., Knapp, D., Nacu, E., Khattak, S., Maden, M, Epperlein, H.H., and Tanaka, E.M. (2009) Cells keep a memory of their tissue origin during axolotl limb regeneration. *Nature,* **460,** 60–65.

63. Alvarado, A.S. (2009) A cellular view of regeneration. *Nature* **460,** 39–40.
64. Bryant, S.V., Endo, T., and Gardiner, D.M. (2002) Vertebrate limb regeneration and the origin of limb stem cells. *Int. J. Dev. Biol.* **46,** 887–896.
65. Snow, M.H. (1977) Myogenic cell formation in regenerating rat skeletal muscle injured by mincing I. A fine structural study. *Anat. Rec.* **188,** 181–199.
66. Slack, J.M.W. (2006) Amphibian muscle regeneration-dedifferentiation or satellite cells? *Trends Cell Biol.* **16,** 273–275.
67. Cameron, J.A., Hilgers, A.R., and Hinterberger, T.J. (1986) Evidence that reserve cells are a source of regenerated adult newt muscle *in vitro. Nature* **321,** 607–610.
68. Tanaka, E.M. (2003) Regeneration: if they can do it, why can't we? *Cell* **113,** 559–562.
69. Stocum, D.L. (2004) Amphibian regeneration and stem cells. *Curr. Top. Microbiol. Immunol.* **280**, 1–70.
70. Tsonis, P.A. (2008) Stem cells and blastema cells. *Curr. Stem Cell Res. Ther.* **3,** 53–54.
71. Maki, M., Suetsugu-Makim, R., Tarui, H., Agatam J., Del Rio-Tsonis, K., and Tsonis, P. (2009) Expression of stem cell pluripotency factors during regeneration in newts. *Dev. Dyn.* **238,** 1613–1616.
72. Oberpriller, J. (1967) A radioautographic analysis of the potency of blastemal cells in the adult newt, *Diemictylus viridescens. Growth* **31,** 251–296.
73. Steen, T.P. (1968) Stability of chondrocyte differentiation and contribution of muscle to cartilage during limb regeneration in the axolotl (*Siredon mexicanum). J. Exp. Zool.* **167,** 49–78.
74. Namenswirth, M. (1974) The inheritance of cell differentiation during limb regeneration in the axolotl. *Dev. Biol.* **41,** 42–56.
75. Ferretti, P. and Brockes, J.P. (1988) Culture of newt cells from different tissues and their expression of a regeneration-associated antigen. *J. Exp. Zool.* **247,** 77–91.
76. Lo, D.C., Allen, F., and Brockes, J.P. (1993) Reversal of muscle differentiation during urodele limb regeneration. *Proc. Natl. Acad. Sci. U. S. A.* **90,** 7230–7234.
77. Kumar, A., Velloso, C., Imokawa, Y., and Brockes, J.P. (2000) Plasticity of retrovirus-labelled myotubes in the newt regeneration blastema. *Dev. Biol.* **218,** 125–136.
78. Okada, T.S. (1996) *Transdifferentiation.* Clarendon, Oxford.
79. Zhou, Q., Brown, J., Kanarek, A., Rajagopal, J., and Melton, D.A. (2008) *In vivo* reprogramming of adult pancreatic exocrine cells to β-cells. *Nature* **455,** 627–632.
80. Belloch, R. (2008) Regenerative medicine: short cut to cell replacement. *Nature* **455**, 604–605.
81. Tsonis, P.A. and Del Rio-Tsonis, K. (2004) Lens and retina regeneration: transdifferentiation, stem cells and clinical applications. *Exp. Eye Res.* **78,** 161–172.
82. Reyer, R.W., Woolfitt, R.A., and Wisthersty, L.I. (1973) Stimulation of lens regeneration from the newt dorsal iris when implanted into the blastema of the regenerating limb. *Dev. Biol.* **32,** 258–281.
83. Echeverri, K. and Tanaka, E.M. (2002) Ectoderm to mesoderm lineage switching during axolotl tail regeneration. *Science* **298,** 1993–1996.
84. Stocum, D.L. (2002) A tail of transdifferentiation. *Science* **298,** 1901–1903.
85. Stocum, D.L. (2006) An overview of regenerative biology and medicine. In *Regenerative Biology and Medicine.* Elsevier, Burlington, MA. Chap. 1.
86. Kawakami, Y., Esteban, R., Raya, M., Kawakami, H., Marti, M., Dubova, I., and Izpisua Belmonte, J. C. (2006) Wnt/beta-catenin signaling regulates vertebrate limb regeneration. *Genes Dev.* **20,** 3232–3247.
87. Yokohama, H.J., Ogino, H., Stoick-Cooper, C.L., Grainger, R.M., and Moon, R. (2007) Wnt/b-catenin signaling has an essential role in the initiation of limb regeneration. *Dev. Biol.* **306,** 170–178.
88. Edwards, R.G. (2008) From embryonic stem cells to blastema and MRL mice. *Reprod. Biomed. Online* **16,** 425–431.
89. Ghosh, S., Roy, S., Sequin, C., Bryant, S.V., and Gardiner, D.M. (2008) Analysis of the expression and function of Wnt-5a and Wnt-5b in developing and regenerating axolotl (*Ambystoma mexicanum)* limbs. *Dev. Growth. Differ.* **50,** 289–297.
90. Simon, H.G., Nelson, C., Goff, D., Laufer, E., Morgan, B.A., and Tabin, C. (1995) Differential expression of myogenic regulatory genes and Msx-1 during dedifferentiation and redifferentiation of regenerating amphibian limbs. *Dev. Dyn.* **202,** 1–12.
91. Kumar, A., Velloso, C., Imokawa, Y., and Brockes, J.P. (2004) The regenerative plasticity of isolated urodele myofibers and its dependence on MSX1. *PloS Biol.* **2,** 1168–1176.
92. Gardiner, D.M. and Bryant, S.V. (1996) Molecular mechanisms in the control of limb regeneration: the role of homeobox genes. *Int. J. Dev. Biol.* **40,** 797–804.
93. Simon, H.-G., Kittappa, R., Khan, P.A., Tsilfidis, C., Liversage, R.A., and Oppenheimer, S. (1997) A novel family of T-box genes in urodele amphibian limb development and regeneration: candidate genes involved in vertebrate forelimb/hindlimb patterning. *Development* **124**, 1355–1366.
94. Lu, J., Jeong, H.-W., Kong, N., Yang, Y., Carroll, J., Luo, H.R., Sillberstein, L.E., Ma, Y., and Chai, L. (2009) Stem cell factor SALL4 represses the transcriptions of PTEN and SALL1 through an epigenetic repressor complex. *PloS One* **4(5)**, e5577.
95. Neff, A.W., King, M.W., Harty, M.W., Nguyen, T., Calley, J., Smith, R.C., and Mescher, L. (2005) Expression of *Xenopus* XlSALL4 during limb development and regeneration. *Dev. Dyn.* **233**, 356–567.
